# The relationship between hope and learning engagement among Chinese university students: the serial mediating roles of generative AI acceptance and self-directed learning

**DOI:** 10.3389/fpsyg.2026.1935139

**Published:** 2026-08-24

**Authors:** Yinshun Zhang, Zimin Wang, Huafu Xu

**Affiliations:** 1 School of Financial Technology and Accounting, Guangdong University of Science & Technology, Dongguan, Guangdong, China; 2 Department of Convergence Lifelong Education, Hanseo University, Seosan, Republic of Korea; 3 School of Computer Science and Information Security, Guilin University of Electronic Technology, Guilin, China; 4 Graduate School of Education, Peking University, Beijing, China; 5 Guangxi Zhuang Autonomous Region Bureau of Big Data Development, Nanning, China

**Keywords:** generative AI acceptance, hope, learning engagement, self-directed learning, university student

## Abstract

The rapid development of generative artificial intelligence (GenAI) has transformed higher education and created new opportunities for student learning. However, the psychological and behavioral factors associated with students’ learning engagement in AI-supported learning environments remain insufficiently understood. Drawing upon Hope Theory, the Technology Acceptance Model (TAM), and Self-directed Learning Theory, this study proposed a serial mediation model to examine the serial mediating roles of generative AI acceptance and self-directed learning in the association between hope and learning engagement. Data were collected from 478 Chinese university students via an online questionnaire. The data were analyzed using SPSS (Version 26.0) and AMOS (Version 24.0), and structural equation modeling (SEM) was employed to evaluate the proposed model. The results showed that hope was positively associated with learning engagement. Moreover, generative AI acceptance and self-directed learning each demonstrated significant indirect associations in the relationship between hope and learning engagement. A significant serial indirect association through generative AI acceptance and self-directed learning was also observed. These findings are consistent with a psychological–technological–behavioral framework linking hope, generative AI acceptance, self-directed learning, and learning engagement in AI-supported learning environments. The study contributes to the application of Hope Theory in the context of generative AI-supported education, advances the growing literature on generative AI acceptance in higher education, and provides practical implications for fostering students’ learning engagement through the effective integration of GenAI and self-directed learning.

## 1 Introduction

Learning engagement has attracted increasing attention in higher education research because it is widely recognized as a critical determinant of students’ academic achievement, persistence, and overall learning success (Fredricks et al., 2004; Kahu, 2013). As a positive and fulfilling learning-related state characterized by vigor, dedication, and absorption, learning engagement reflects the extent to which students invest cognitive, emotional, and behavioral resources in learning activities (Schaufeli et al., 2002). Extensive evidence has demonstrated that engaged students are more likely to achieve superior academic outcomes, exhibit greater persistence in challenging learning tasks, and experience higher levels of learning satisfaction (Bond et al., 2020). Consequently, identifying the factors that influence learning engagement has become a major concern for educational researchers and practitioners.

The rapid development of generative artificial intelligence (GenAI) has fundamentally transformed learning environments in higher education. Since the introduction of large language models such as ChatGPT, students have increasingly utilized GenAI technologies for information retrieval, content generation, problem-solving, and personalized academic support (Dwivedi et al., 2023). Recent studies suggest that GenAI can enhance learning efficiency, foster personalized learning experiences, and support students’ academic development (Chiu et al., 2023). Furthermore, a recent meta-analysis reported that GenAI positively influences students’ motivation and learning engagement across a variety of educational contexts (Xia et al., 2025). However, despite the growing adoption of GenAI in higher education, existing research has primarily focused on technological characteristics and learning outcomes. At the same time, relatively little attention has been paid to the psychological mechanisms through which students become actively engaged in AI-supported learning environments.

Positive psychology provides an important perspective for understanding students’ engagement in contemporary learning contexts. Among various positive psychological resources, hope has emerged as a particularly influential construct. According to Hope Theory, hope consists of agency thinking, which reflects individuals’ motivation to pursue goals, and pathways thinking, which represents their perceived ability to identify routes for achieving those goals (Snyder, 2002). Previous studies have consistently demonstrated that hope is positively associated with academic achievement, self-efficacy, wellbeing, and learning engagement (Azila-Gbettor et al., 2021; Feldman and Kubota, 2015). More recently, Gallagher (2023) argued that hope functions as a foundational psychological resource that facilitates adaptive functioning and sustained motivation in educational settings. However, empirical evidence on how to influence learning engagement remains limited in technology-mediated or GenAI-supported contexts.

Beyond psychological resources, successful learning in the age of artificial intelligence increasingly depends on students’ acceptance of educational technologies and their ability to regulate their own learning. Drawing on the Technology Acceptance Model (Davis, 1989), students who perceive GenAI as useful and beneficial are more likely to adopt it as a learning resource. At the same time, effective use of GenAI requires self-directed learning, defined as learners’ ability to independently plan, monitor, and evaluate their learning processes (Garrison, 1997; Knowles, 1975). Previous studies suggest that GenAI can support self-directed learning by providing personalized guidance and adaptive feedback, thereby encouraging greater learner autonomy (Chiu et al., 2023). Therefore, generative AI acceptance and self-directed learning may represent important mechanisms through which psychological resources are translated into learning engagement.

Despite the growing literature on hope, technology acceptance, self-directed learning, and GenAI-supported education, several important research gaps remain. First, although hope has been widely recognized as an important predictor of educational outcomes, its relationship with generative AI acceptance remains largely unexplored. Second, limited research has examined how generative AI acceptance influences self-directed learning within AI-supported learning environments. Third, previous studies have typically investigated psychological, technological, and behavioral factors separately, resulting in a fragmented understanding of student engagement in the era of generative artificial intelligence. To date, little empirical research has integrated these perspectives into a single explanatory framework.

To address these gaps, the present study integrates Hope Theory (Snyder, 2002), the Technology Acceptance Model (Davis, 1989), and self-directed learning theory (Garrison, 1997; Knowles, 1975) to develop a comprehensive framework explaining learning engagement in the era of generative artificial intelligence. Specifically, a serial mediation model is proposed in which hope is associated with learning engagement through generative AI acceptance and self-directed learning. By integrating psychological, technological, and behavioral perspectives, this study contributes to a more comprehensive understanding of student engagement in AI-supported learning and provides practical implications for the effective integration of generative AI in higher education.

## 2 Literature review and research hypotheses

### 2.1 Theoretical framework

**FIGURE 1:**
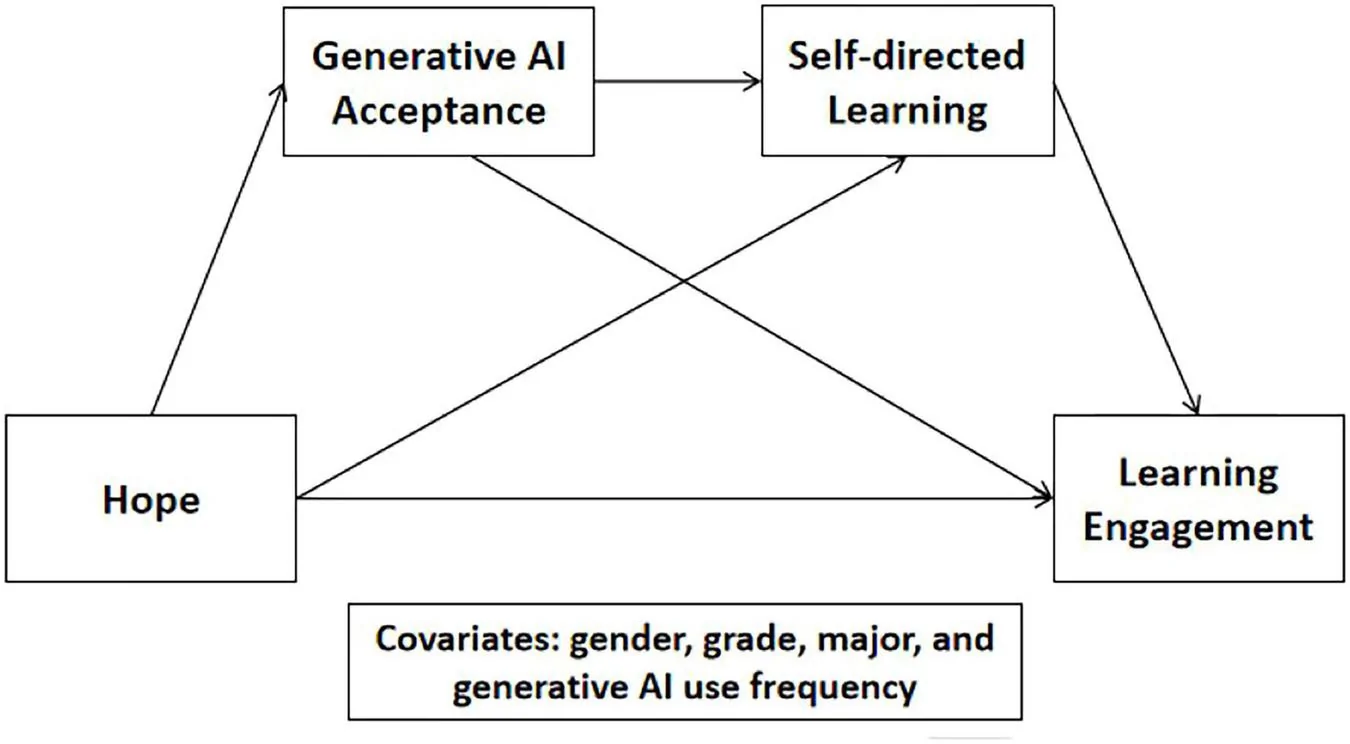
Research hypothesis model.

This study proposes a psychological–technological–behavioral framework to explain the relationships among hope, university students’ learning engagement, and the proposed mediating variables in the context of generative artificial intelligence (GenAI), as depicted in Figure 1. Drawing upon Hope Theory (Snyder et al., 1991), the Technology Acceptance Model (TAM) (Davis, 1989), and self-directed learning theory (Garrison, 1997; Knowles, 1975), the framework posits that hope serves as a psychological resource that is associated with students’ acceptance of GenAI, self-directed learning, and learning engagement. Specifically, students with higher levels of hope may be more likely to perceive GenAI as a valuable learning resource and report greater acceptance of AI-assisted learning tools. Greater acceptance of GenAI is expected to be associated with higher levels of self-directed learning, as learners may use AI-supported feedback and personalized assistance to plan, monitor, and regulate their learning more effectively. In turn, self-directed learning is expected to be positively associated with greater cognitive, emotional, and behavioral involvement in academic activities. Accordingly, the proposed framework conceptualizes learning engagement as being associated with the interaction among psychological resources (hope), technological acceptance (GenAI acceptance), and behavioral regulation (self-directed learning), providing a comprehensive theoretical perspective on the relationships among these constructs in AI-supported educational environments.

### 2.2 The relationship between hope and learning engagement

Learning engagement is conceptualized in the present study as a multidimensional construct encompassing students’ behavioral involvement, emotional connection, and cognitive investment in academic activities (Fredricks et al., 2004). Within this framework, the relationship between hope and learning engagement is theoretically coherent and empirically well-grounded.

Empirically, a previous research analysis by Marques et al. (2011) demonstrated that hope maintains a moderate, positive relationship with academic achievement and is consistently associated with student engagement assets across educational levels. Gallagher et al. (2017) further showed, in a 4-year longitudinal study of college students, that hope uniquely predicted academic persistence, retention, and cumulative GPA beyond the contributions of self-efficacy and engagement measured independently. More recently, Zhang et al. (2025) demonstrated that hope positively associated with learning engagement, with the pathway from hope to engagement being both theoretically grounded in self-determination theory and statistically significant in structural equation modeling. Liang et al. (2023) further confirmed that psychological capital constructs, including hope, are associated with engagement outcomes in blended learning university courses. Students with higher hope are better equipped to navigate these uncertainties because their agency thinking sustains goal commitment under pressure and their pathways thinking enables proactive adaptation to new technological affordances. In the specific context of the GenAI era, where students face unprecedented uncertainty about the nature and value of their learning, hope’s role as a stabilizing, forward-oriented psychological resource may be particularly salient in sustaining engagement. Accordingly, the following hypothesis is proposed:

**H1: Hope is positively associated with learning engagement**.

### 2.3 The mediating role of generative AI acceptance

Generative AI acceptance refers to students’ willingness to adopt and intentionally integrate GenAI tools into their academic learning activities, which is measured via six dimensions: perceived usefulness, perceived ease of use, perceived risk, technological anxiety, social impact, and intention to use. Following the TAM framework (Davis, 1989), acceptance is driven by beliefs about the technology’s usefulness for goal achievement and perceptions about the effort required to use it effectively. As GenAI tools such as ChatGPT and related large language model applications become increasingly prevalent in higher education, students’ acceptance of these tools has emerged as a key determinant of their learning behavior (Chan and Hu, 2023; Guo et al., 2025).

Two theoretical linkages anchor this mediating pathway. First, hope is expected to positively associated with GenAI acceptance. Students endowed with a high agency thinking approach new technologies from a goal-aligned orientation: they evaluate whether a technology can serve as a reliable pathway toward their academic objectives rather than as a source of distraction or risk. Their pathways thinking further inclines them to view GenAI tools as flexible, expandable resources for problem-solving, aligning with Snyder’s (2002) observation that high-hope individuals are more likely to engage with novel means in service of valued goals. Cabero-Almenara et al. (2024) demonstrated that constructivist-oriented learners who emphasize active goal pursuit show greater openness to adopting AI tools, consistent with the hope-acceptance linkage proposed here. Students with stronger psychological goal-directedness are more likely to perceive AI tools as goal-aligned resources rather than threats to academic autonomy.

Second, once students accept GenAI tools and integrate them into their study routines, learning engagement is expected to increase. Guo et al. (2025) analyzed undergraduate students in China and found that GenAI use in academic tasks is associated with greater cognitive and emotional engagement. Liang et al. (2023) similarly demonstrated that student–GenAI interaction positively associated with learning achievement through engagement-related mediators. Liang and Reiss (2025) further found that positive student attitudes toward AI, a construct closely related to acceptance, were associated with higher learning engagement through perceived autonomy and learning enjoyment. These findings collectively support the proposition that GenAI acceptance, by expanding students’ perceived repertoire of learning strategies and providing on-demand cognitive support, stimulates deeper and more sustained engagement.

Together, these arguments support a mediated pathway from hope to learning engagement via GenAI acceptance. The following hypotheses are proposed:

**H2: Hope is positively associated with generative AI acceptance.**

**H3: Generative AI acceptance is positively associated with learning engagement.**

**H4: Generative AI acceptance mediates the positive relationship between hope and learning engagement.**

### 2.4 The mediating role of self-directed learning

Self-directed learning (SDL) describes the extent to which learners proactively take initiative in diagnosing their learning needs, formulating learning goals, selecting strategies and resources, and evaluating their own progress (Knowles, 1975). As an active behavioral orientation, SDL represents a concrete expression of the internal motivational states that students bring to academic tasks. Students who demonstrate high SDL are more likely to engage deeply with course content, monitor their own understanding, and sustain behavioral investment in learning over time, all of which are constitutive features of learning engagement.

Hope Theory directly maps onto the psychological substrate of SDL. Agency thinking, the motivational energy that drives students toward their goals, corresponds to the self-initiation and desire-for-learning dimensions of SDL: students who believe they can successfully pursue their goals are more likely to proactively seek out learning resources, initiate study strategies, and persist in self-directed activities without external coercion. Pathways thinking, the capacity to generate and revise goal-directed plans, corresponds to the self-management and self-control dimensions of SDL: students who can flexibly plan around obstacles are better equipped to organize their learning environments, adjust strategies when initial approaches fall short, and maintain progress toward learning objectives. Consistent with this reasoning, Zhang et al. (2025) confirmed that hope positively associated with behavioral engagement through autonomous motivational mechanisms, and Liang et al. (2023) demonstrated that self-directed learning behaviors mediate between psychological resources and academic outcomes in university contexts.

Self-directed learning, in turn, is strongly linked to learning engagement. Numerous studies have established that students who demonstrate higher levels of self-directed learning show deeper behavioral, cognitive, and emotional engagement with academic tasks (Xu et al., 2024; Yang, 2025). The self-orientation ability and ability to apply learning strategies dimension of SDL is associated with greater behavioral engagement; the self-monitoring ability dimension corresponds to emotional engagement; and the self-control dimension maps onto cognitive engagement. In Hua et al.’s (2024) study of university students in blended learning contexts, SDL experience was positively related to deep learning approaches and overall learning satisfaction, pathways through which engagement is substantiated.

In sum, hope is expected to stimulate self-directed learning behavior by channeling the student’s goal-directed motivation into proactive learning initiation and strategic planning; and SDL, in turn, is expected to translate this motivational orientation into sustained and multidimensional learning engagement. Accordingly, the following hypotheses are proposed:

**H5: Hope is positively associated with self-directed learning.**

**H6: Self-directed learning is positively associated with learning engagement.**

**H7: Self-directed learning mediates the positive relationship between hope and learning engagement.**

### 2.5 The dual mediating roles of generative AI acceptance and self-directed learning

In GenAI-integrated educational contexts, when students accept and habitually use GenAI tools, these tools can serve as cognitive scaffolds that amplify the capacity for self-directed learning in two ways. First, GenAI tools automate routine cognitive tasks, such as information retrieval, text summarization, and initial draft generation, thereby releasing students’ cognitive bandwidth for higher-order self-regulatory processes, including goal-setting, metacognitive monitoring, and strategic resource allocation. Xu et al. (2025) confirmed empirically that meta-cognitive support in GenAI environments significantly associated with self-regulated learning, and Lan and Zhou’s (2025) systematic review found that AI applications in higher education positively support learners’ self-regulation through personalized feedback and adaptive guidance. Second, GenAI tools provide on-demand, personalized learning support that enables students to become more autonomous and self-directed in their learning trajectories: students who accept and engage with AI tools gain access to immediate feedback, tailored explanations, and flexible learning pathways that reinforce SDL dispositions (Cabero-Almenara et al., 2024; Xu et al., 2025).

Thus, the proposed sequential framework posits that students with higher hope levels may exhibit greater acceptance of generative AI, as hopeful individuals tend to perceive available resources as viable pathways to goal attainment. Meanwhile, higher acceptance of generative AI may correlate with elevated self-directed learning levels, given that students with positive perceptions of AI technologies are more inclined to integrate them into autonomous learning processes. Self-directed learning is further hypothesized to have a positive association with learning engagement, as self-regulated and autonomous learners typically demonstrate higher investment in learning activities. Collectively, these relationships underpin a theoretically grounded sequence linking hope, generative AI acceptance, self-directed learning, and learning engagement. Accordingly, the following hypotheses are proposed:

**H8: Generative AI acceptance is positively associated with self-directed learning.**

**H9: Generative AI acceptance and self-directed learning sequentially mediate the relationship between hope and learning engagement.**

## 3 Research methods

### 3.1 Participants and procedures

This study employed a convenience sampling strategy to recruit undergraduate students from public and private universities across 32 provincial-level administrative regions in mainland China. Data were collected via the Wenjuanxing online survey platform between December 2024 and January 2025. Invitation messages containing an informed consent form and a survey link were distributed electronically through university instructors and student counselors. The invitation explained the study purpose, eligibility criteria, estimated completion time, the voluntary nature of participation, confidentiality protection, and participants’ right to withdraw at any time without penalty. To minimize duplicate responses, the survey platform was configured to restrict multiple submissions from the same device.

A total of 622 questionnaires were initially collected. Data quality was ensured through predefined screening procedures. Specifically, 114 questionnaires completed in less than 100 s were excluded because they were considered insufficient for thoughtful completion, and 30 questionnaires exhibiting inattentive response patterns (identical responses across items) were also removed. After data screening, 478 valid questionnaires were retained for subsequent analyses. The final sample size exceeded commonly recommended thresholds for covariance-based structural equation modeling (CB-SEM), providing adequate statistical power for estimating the proposed measurement and structural models.

Among the 478 participants, 199 were male (41.6%), and 279 were female (58.4%). Regarding grade, 137 (28.7%) were first-year students, 177 (37.0%) were second-year students, 123 (25.7%) were third-year students, and 41 (8.6%) were fourth-year students. In terms of academic discipline, 338 participants (70.7%) were enrolled in humanities and social sciences programmes, whereas 140 (29.3%) were enrolled in natural sciences programmes. Concerning the frequency of generative AI use, 23 students (4.8%) reported never using generative AI, 121 (25.3%) reported rare use, 226 (47.3%) reported occasional use, 90 (18.8%) reported frequent use, and 18 (3.8%) reported very frequent use. Although approximately 30% of participants reported never or rarely using generative AI, the GAA scale primarily assessed perceptions and behavioral intentions rather than actual usage behavior. Participants who had limited experience were instructed to respond based on their understanding, observations, or limited exposure to widely available generative AI tools (e.g., ChatGPT, DeepSeek).

Gender, grade, major, and GenAI use frequency were included as control variables because previous studies have suggested that demographic characteristics may influence students’ learning engagement. Since the above variables are all categorical variables, they were coded as dummy variables before being included in the structural equation model (SEM). These variables were controlled in the structural model by specifying direct paths to learning engagement.

### 3.2 Measuring instruments

#### 3.2.1 Hope

Originating from the scale designed by Snyder (2002), this scale consists of eight items in total, with items such as “I can think of many ways to get out of a jam.” It’s a five-point Likert scale ranging from “Strongly disagree” to “Strongly agree.” An increasing score represents stronger hope. Given the scale’s clear two-dimensional structure, the internal-consistency approach was adopted (Little et al., 2002), with each theoretical dimension serving directly as one parcel indicator, yielding two parcels (Agency and Pathways) for use in CFA and SEM analyses. In this study, the Cronbach’s α was 0.931.

#### 3.2.2 Generative AI acceptance

Generative AI acceptance (GAA) was measured using a revised version of the Technology Acceptance Model (TAM)-based scale adapted by Zhang (2023) and originally grounded in Davis’s (1989) framework. In the present study, generative AI acceptance refers to students’ overall acceptance of generative AI as a learning tool. The scale includes six dimensions: perceived usefulness, Perceived ease of use, Perceived risk, Technological anxiety, Social impact, and Intention to use. The original instrument consisted of 17 items (e.g., “Generative AI tools help me save time when searching for information”) assessing students’ acceptance of generative AI technologies. All items were rated on a five-point Likert scale ranging from 1 (strongly disagree) to 5 (strongly agree), with higher scores indicating higher levels of generative AI acceptance. Following the psychometric screening criteria recommended by Hair et al. (2022), two items (item7, item 12) with severe cross-loadings and three items (item 6, item 8, item 14) with weak standardized factor loadings were removed, resulting in a refined 12-item scale. To reduce model complexity and enhance parameter estimation stability, a factor-based parceling strategy was applied based on Exploratory Factor Analysis (EFA) results (Kline, 2023). The 12 retained items were aggregated into three empirical parcels representing the underlying condensed dimensional structure of the construct. A reliability analysis of the refined 12-item scale yielded a Cronbach’s α of 0.906.

#### 3.2.3 Self-directed learning

Self-directed learning (SDL) was measured using the scale adapted by Cai (2023). The instrument assesses university students’ self-directed learning abilities and includes items such as “I study according to my own learning plan” and “I have confidence in my learning ability.” The original scale consisted of 14 items. All items were rated on a five-point Likert scale ranging from 1 (strongly disagree) to 5 (strongly agree), with higher scores indicating higher levels of self-directed learning. Item screening identified two items with substantial cross-loadings (item 7, item 8), which were subsequently removed, leaving 12 items for analysis. Based on EFA results and to maintain optimal model parsimony, a factor-based parceling strategy was deployed (Kline, 2023). The 12 retained items were aggregated into two major parcels based on the empirical factor structure, which served as latent indicators in the subsequent SEM analyses. A reliability analysis of the final 12-item scale yielded a Cronbach’s α of 0.961.

#### 3.2.4 Learning engagement

Learning engagement (LE) was measured using the scale adapted by Chi (2017), which was derived from the Student Engagement Scale developed by Lam et al. (2012). The instrument assesses students’ engagement in learning activities across behavioral, emotional, and cognitive dimensions and includes items such as “At school, I strive to perform well” and “When studying, I decide which key points to focus on rather than reading broadly.” The original scale consisted of 15 items. All items were rated on a five-point Likert scale ranging from 1 (strongly disagree) to 5 (strongly agree), with higher scores indicating higher levels of learning engagement. During item screening, three items (item 1, item 2,item 3) with substantial cross-loadings and one reverse-worded item (item 7) exhibiting poor psychometric performance were removed (Hair et al., 2022), yielding 11 retained items. The three theoretical engagement dimensions were each represented by one parcel indicator, resulting in three parcels that reflect the construct’s theoretically distinct subdimensions (Little et al., 2002). Reliability analysis based on the final 11-item scale yielded a Cronbach’s α coefficient of 0.954.

#### 3.2.5 Exploratory factor analysis and scale refinement

Prior to confirmatory factor analysis (CFA), exploratory factor analysis (EFA) was conducted to examine the underlying factor structure of the adapted scales. Principal component analysis with Kaiser-normalized varimax rotation was employed to identify the latent factor structure, and factors with eigenvalues greater than 1.0 were retained.

Item refinement followed predefined statistical and theoretical criteria. During EFA, items were considered for removal if they met one or more of the following conditions: (a) a primary factor loading below 0.50; (b) substantial cross-loadings, defined as a difference of less than 0.20 between the primary and secondary loadings; or (c) conceptual redundancy with other items. During CFA, additional items were removed only when they simultaneously exhibited relatively low standardized factor loadings, substantial localized strain indicated by large modification indices, and conceptual overlap with other items within the same latent construct. Accordingly, item refinement was conducted sequentially and conservatively, with each deletion guided by both empirical evidence and theoretical justification rather than model-fit improvement alone.

Following scale refinement, the final GAA scale consisted of three dimensions: perceived usefulness, technology anxiety, and behavioral intention to use. Together, these dimensions capture students’ cognitive evaluation of generative AI, emotional responses toward the technology, and their willingness to adopt it for learning purposes. The final SDL scale consisted of two dimensions: Self-orientation and learning ability, and self-monitoring and evaluation ability. The final LE scale still maintained the three dimensions of theory.

Because EFA, CFA, and SEM were conducted using the same sample, it should be regarded as exploratory rather than definitive. Therefore, the revised factor structure warrants further cross-validation using independent samples in future research.

### 3.3 Data analysis

Data were analyzed using SPSS 26.0 and AMOS 24.0 in three steps. First, preliminary analyses were conducted, including normality tests, descriptive statistics, exploratory factor analysis, and Pearson correlations among the key variables. Second, confirmatory factor analysis, reliability, and validity evaluations were carried out, and common method bias was evaluated through the unmeasured latent method construct (ULMC) technique before hypothesis testing. Third, the hypothesized model was tested with maximum likelihood estimation in AMOS 24.0 to examine direct paths. Bootstrapping procedures (5,000 resamples) were then employed, and bias-corrected 95% confidence intervals were computed to assess simple mediation and chain mediation effects. Effects were considered significant if the intervals did not include 0. Thereby, determining whether generative AI acceptance and self-directed learning serially transmitted the effect of hope on learning engagement.

### 3.4 Common method bias test

Because all variables were measured using self-report questionnaires, common method bias (CMB) could not be completely ruled out. To reduce potential method bias, participants were assured of anonymity and confidentiality before completing the survey. Following the recommendations of Podsakoff et al. (2003), an unmeasured latent method construct (ULMC) approach was employed to assess the potential influence of CMB.

The theoretical measurement model demonstrated a satisfactory fit to the data (χ2/df = 2.024, CFI = 0.991, TLI = 0.985, RMSEA = 0.046, SRMR = 0.035). Subsequently, a latent common method factor was added and allowed to load on all observed indicators. The method-factor model also demonstrated acceptable fit (χ2/df = 2.042, CFI = 0.992, TLI = 0.985, RMSEA = 0.047, SRMR = 0.034). The changes in fit indices between the two models were relatively small (ΔCFI = 0.001, ΔTLI = 0, ΔRMSEA = 0.001, ΔSRMR = 0.001) and remained below the commonly recommended thresholds (CFI/TLI < 0.05; RMSEA/SRMR < 0.015). Therefore, common method bias was unlikely to have substantially affected the findings of the present study.

## 4 Research results

### 4.1 Descriptive statistics and correlations

The results of the Pearson correlation analysis are presented in Table 1. GAA showed a significant positive correlation with hope (*r* = 0.372, *p* < 0.01), LE (*r* = 0.482, *p* < 0.01), SDL (*r* = 0.425, *p* < 0.01). Hope was positively correlated with LE (*r* = 0.671, *p* < 0.01), and SDL (*r* = 0.598, *p* < 0.01). LE was positively correlated with SDL (*r* = 0.668, *p* < 0.01).

**TABLE 1:** Descriptive statistics and Pearson correlations.

Construct	GAA	Hope	LE	SDL
GAA	1	–	–	–
Hope	0.372**	1	–	–
LE	0.482**	0.671**	1	–
SDL	0.425**	0.598**	0.668**	1
M	3.609	3.580	3.737	3.693
SD	0.537	0.675	0.634	0.730
Skewness	0.539	0.346	0.04	−0.172
Kurtosis	0.409	−0.075	0.381	0.533

**P < 0.01. GAA, generative AI acceptance; SDL, self-directed learning; LE, learning engagement.

As a result of the descriptive statistical analysis, the mean values of all four major variables exceeded the median value of three points, with learning engagement showing the highest mean among them. Furthermore, the skewness and kurtosis of all variables were within acceptable ranges, indicating that the sample data of this study followed a normal distribution. Multicollinearity was assessed by examining variance inflation factors (VIF) for all predictor variables. All VIF values were below 3.0 (range: 1.253–1.68), well within the acceptable threshold of 5.0, indicating that multicollinearity is not a concern in the present study.

### 4.2 Measurement model assessment

Before evaluating the measurement model, it should be noted that the original measurement model comprised 43 observed indicators across the four latent constructs. Estimating all item-level indicators simultaneously would substantially increase model complexity and the number of free parameters, potentially reducing estimation stability and model parsimony in covariance-based structural equation modeling (CB-SEM). Therefore, following the recommendations of Little et al. (2002), an item-parceling strategy was adopted prior to structural model estimation. Specifically, Hope and Learning Engagement were parceled according to their theoretically defined dimensions, whereas Generative AI Acceptance and Self-directed Learning were parceled based on the factor structure identified through exploratory factor analysis. Consequently, the final measurement model consisted of 10 observed indicators representing four latent constructs (Hope = 2 parcels; Generative AI Acceptance = 3 parcels; Self-directed Learning = 2 parcels; Learning Engagement = 3 parcels).

As shown in Table 2, the Average Variance Extracted (AVE) values of the four core constructs spanned from 0.536 to 0.839, exceeded the threshold of 0.5, and all Composite Reliability (CR) values ranged from 0.766 to 0.912, surpassing the recommended value of 0.7, which indicates satisfactory convergent validity and composite reliability. To evaluate discriminant validity, the Fornell–Larcker Criterion and the Heterotrait–Monotrait (HTMT) ratio of correlations were utilized. As presented in Tables 2, 3, the square roots of AVE for all constructs exceeded their inter-construct correlations, and all HTMT values were below the conservative threshold of 0.90, indicating satisfactory discriminant validity.

**TABLE 2:** Reliability, convergent validity, and latent correlations.

Construct	CR	AVE	Hope	GAA	SDL	LE
Hope	0.912	0.839	**0.916**	–	–	–
GAA	0.766	0.536	0.412***	**0.732**	–	–
SDL	0.888	0.800	0.666***	0.463***	**0.894**	–
LE	0.910	0.772	0.730***	0.533***	0.733***	**0.879**

***P < 0.001. GAA, Generative AI acceptance; SDL, self-directed learning; LE, learning engagement. Diagonal values (bold) represent the square roots of the AVE; off-diagonal values are latent correlations estimated from the final measurement model.

**TABLE 3:** Heterotrait–Monotrait ratio (HTMT) analysis.

Construct	Hope	GAA	SDL	LE
Hope	–	–	–	–
GAA	0.452	–	–	–
SDL	0.660	0.522	–	–
LE	0.728	0.586	0.739	–

GAA, generative AI acceptance; SDL, self- directed learning; LE, learning engagement.

### 4.3 Structural model and hypothesis testing

To examine whether the observed associations among the study variables were consistent with the proposed theoretical framework, the hypothesized structural model was evaluated using structural equation modeling (SEM) in AMOS. As shown in Table 4, the structural model demonstrated an acceptable fit to the data. Gender, grade, major, and generative AI use frequency were included as control variables because these characteristics may be associated with university students’ learning engagement.

**TABLE 4:** Structural model fit indices.

Fit index	χ^2^	df	χ^2^/df	CFI	TLI	RMSEA	SRMR
Suggested value	–	–	1–5	>0.900	>0.900	<0.080	<0.080
Value of this study	251.692	71	3.545	0.946	0.930	0.073	0.072

**FIGURE 2:**
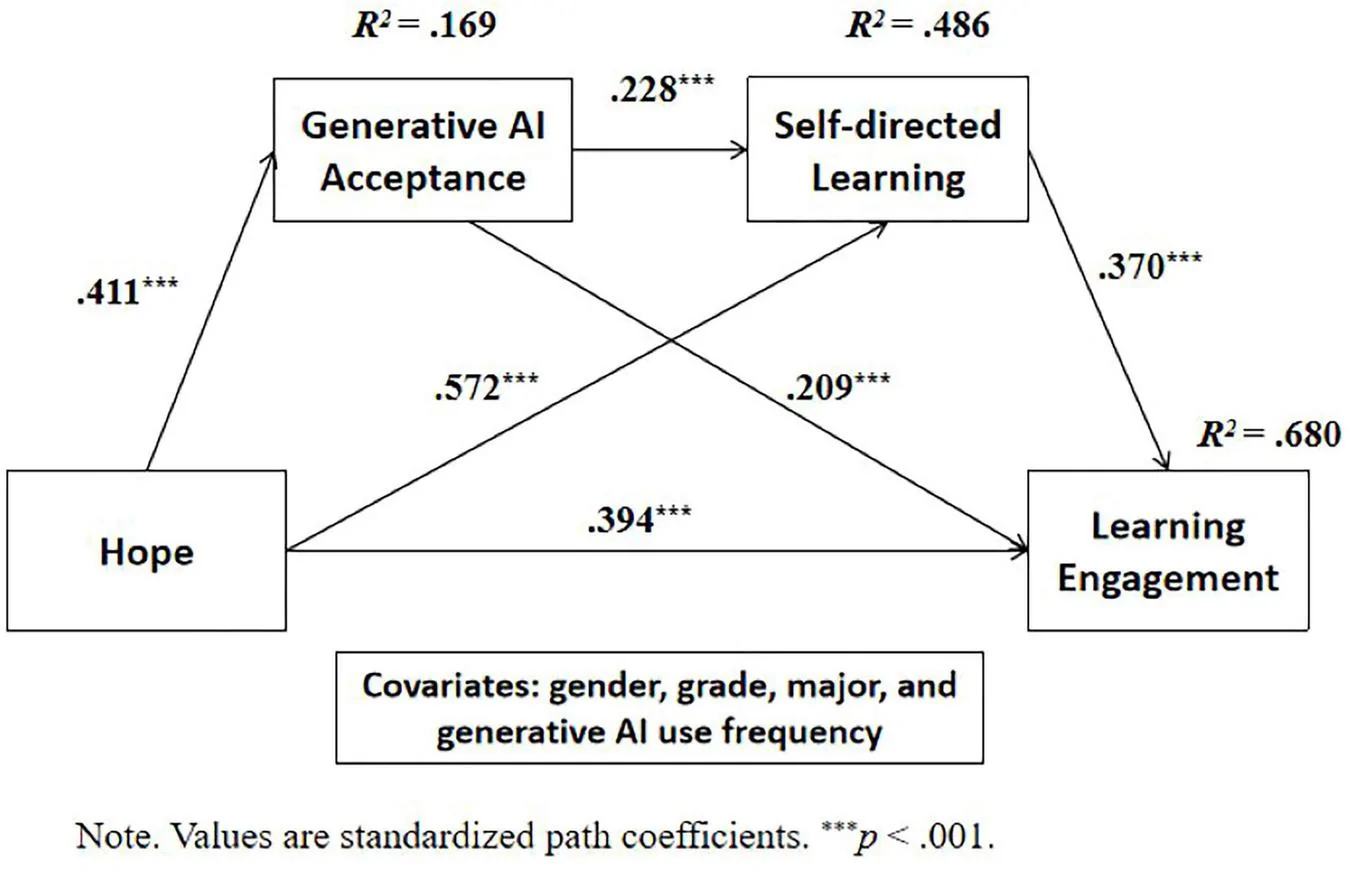
Path diagram of the structural equation model. Values are standardized path coefficients. ***p < 0.001.

The standardized path coefficients are presented in Figure 2 and Table 5. Overall, the observed associations were consistent with the hypothesized structural model. Hope was positively associated with generative AI acceptance (β = 0.411, *p* < 0.001), self-directed learning (β = 0.572, *p* < 0.001), and learning engagement (β = 0.394, *p* < 0.001). In addition, generative AI acceptance was positively associated with self-directed learning (β = 0.228, *p* < 0.001) and learning engagement (β = 0.209, *p* < 0.001), while self-directed learning was positively associated with learning engagement (β = 0.370, *p* < 0.001). Accordingly, all hypothesized associations were statistically significant.

**TABLE 5:** Structural model path estimates and hypothesis testing.

Hypothesis	Path	B	β	SE	CR	*P*	Decision
H1	Hope → LE	0.372	0.394	0.046	8.088	<0.001	Supported
H2	Hope → GAA	0.333	0.411	0.043	7.811	<0.001	Supported
H3	GAA → LE	0.244	0.209	0.047	5.154	<0.001	Supported
H5	Hope → SDL	0.584	0.572	0.051	11.451	<0.001	Supported
H6	SDL → LE	0.342	0.370	0.046	7.459	<0.001	Supported
H8	GAA → SDL	0.287	0.228	0.058	4.940	<0.001	Supported

GAA, generative AI acceptance; SDL, self-directed learning; LE, learning engagement. B, unstandardized estimate; β, standardized estimate; SE, standard error; CR, critical ratio.

In addition, the structural model accounted for 16.9% of the variance in generative AI acceptance (R^2^ = 0.169), 48.6% of the variance in self-directed learning (R^2^ = 0.486), and 68.0% of the variance in learning engagement (R^2^ = 0.680). These findings indicate that the proposed model explained a substantial proportion of the observed variance in learning engagement.

### 4.4 Bootstrap analysis of mediation effects

To further examine the hypothesized indirect pathways, a bias-corrected bootstrap procedure with 5,000 resamples was conducted. An indirect pathway was considered statistically significant when its 95% bias-corrected confidence interval (CI) did not include zero. The bootstrap results are presented in Table 6.

**TABLE 6:** Bootstrap estimates of indirect and serial mediation effects.

Effect type	Pathway	B	β	Boot SE	95% LLCI	95% ULCI
Direct effect	Hope → LE	0.372	0.394	0.067	0.235	0.484
Indirect effect	Hope→GAA→LE	0.081	0.086	0.029	0.027	0.141
	Hope→SDL→LE	0.200	0.212	0.055	0.107	0.322
	Hope→GAA→SDL→LE	0.033	0.035	0.012	0.019	0.065
Total effect	Hope → LE	0.686	0.727	0.044	0.591	0.765

GAA, generative AI acceptance; SDL, self-directed learning; LE, learning engagement.

The results showed that the indirect pathway through generative AI acceptance, the indirect pathway through self-directed learning, and the sequential indirect pathway through generative AI acceptance and self-directed learning were all statistically significant, as their 95% confidence intervals did not include zero. These findings were consistent with H4, H7, and H9.

The total indirect association accounted for approximately 45.8% of the total association between hope and learning engagement, indicating that a substantial proportion of the observed association was accounted for by the indirect pathways specified in the proposed model. In addition, the sequential indirect pathway was statistically significant, suggesting that the observed association between hope and learning engagement was consistent with the hypothesized sequence involving generative AI acceptance and self-directed learning.

### 4.5 Alternative model and sensitivity analysis

To examine the robustness of the proposed sequential mediation model, two theoretically plausible competing models were tested. Specifically, a reversed serial mediation model (Hope → Self-directed Learning → Generative AI Acceptance → Learning Engagement) and a parallel mediation model, in which generative AI acceptance and self-directed learning operated as parallel mediators, were estimated.

The alternative model analysis showed that all three structural models demonstrated acceptable overall fit. The reversed serial mediation model yielded model-fit indices comparable to those of the hypothesized model, with only negligible differences across the global fit statistics. This finding suggests that the present cross-sectional data do not provide sufficient evidence to distinguish the temporal ordering of the two mediators based solely on model fit. Accordingly, the hypothesized model was retained because its sequential pathway is more consistent with the theoretical framework underpinning this study. Specifically, Hope Theory posits that hopeful individuals are more likely to adopt adaptive learning resources and strategies, the Technology Acceptance Model provides the theoretical basis for understanding students’ acceptance of generative AI, and Self-directed Learning Theory suggests that technology acceptance facilitates learners’ autonomous learning behaviors, which in turn are associated with higher levels of learning engagement. Therefore, the proposed sequence was determined primarily by theoretical considerations rather than by minor differences in statistical model fit.

To further examine the robustness of the findings, a sensitivity analysis was conducted by excluding participants who reported never using generative AI (*N* = 455). The structural model was re-estimated using the reduced sample. The overall pattern of results remained substantively unchanged. The measurement model continued to demonstrate acceptable fit, and all direct, indirect, and serial mediation associations remained statistically significant with only trivial changes in the estimated coefficients and confidence intervals. These research findings demonstrate that the proposed model is robust, and the observed correlations remain consistent regardless of whether participants lacking experience with generative AI are included. Detailed CFA, correlation, discriminant validity, and SEM outputs, as well as scale refinement details, alternative model comparisons and sensitivity analysis, are presented in Supplementary Tables 1–3.

## 5 Discussion

The present study examined the associations among hope, generative AI acceptance, self-directed learning, and learning engagement by testing a theoretically informed serial mediation model. Consistent with the proposed framework, hope was positively associated with learning engagement both directly and indirectly through generative AI acceptance and self-directed learning. The findings further indicated that generative AI acceptance and self-directed learning jointly represented a statistically significant serial indirect pathway linking hope and learning engagement. Given the cross-sectional design, these findings should be interpreted as associations that are consistent with the proposed theoretical framework rather than evidence of causal or temporal relationships. The findings are discussed below.

### 5.1 Hope as an important psychological resource for learning engagement

Consistent with Hypothesis 1, hope was positively associated with learning engagement. This finding is consistent with Hope Theory, which posits that individuals with high levels of hope possess both agency thinking and pathways thinking that enable them to pursue goals persistently despite obstacles (Snyder, 2002). The present finding is consistent with previous research reporting positive associations between hope and a range of educational outcomes, including academic achievement, self-efficacy, psychological wellbeing, and learning engagement (Azila-Gbettor et al., 2021; Feldman and Kubota, 2015).

More recently, Gallagher (2023) argued that hope functions as a key psychological resource that promotes adaptive functioning across educational contexts. Similarly, research conducted in AI-supported learning environments has shown that positive psychological resources contribute to students’ willingness to engage with emerging technologies and sustain learning efforts (Wang and Wu, 2025). Recent meta-analytic evidence further suggests that students with stronger motivational resources tend to exhibit higher engagement when learning with GenAI-supported tools (Xia et al., 2025).

The present study contributes to this growing body of literature by showing that the positive association between hope and learning engagement is also evident in the context of generative AI-supported learning. Compared with traditional learning environments, AI-supported learning often requires learners to independently evaluate information quality and make continuous learning decisions. Hopeful students may be more capable of navigating these challenges because they actively seek pathways to goal attainment and maintain motivation when difficulties arise (Snyder, 2002).

### 5.2 The mediating role of generative AI acceptance

Consistent with H4, generative AI acceptance demonstrated a statistically significant indirect association between hope and learning engagement. This finding is consistent with the theoretical assumptions of the Technology Acceptance Model (Davis, 1989), suggesting that students’ acceptance of generative AI is associated with the relationship between positive psychological resources and learning engagement.

Previous studies have consistently reported that students’ perceptions of GenAI usefulness and ease of use significantly predict their intention to adopt AI technologies in educational settings (Shahzad et al., 2025; Strzelecki, 2024; Zhao et al., 2024). Moreover, students who positively perceive AI technologies tend to report higher learning motivation, engagement, and satisfaction (Lv et al., 2025; Wang and Wu, 2025).

An important contribution of this study is the identification of hope as a psychological factor associated with GenAI acceptance. Previous TAM studies have primarily focused on technological variables, whereas the current findings suggest that students’ psychological strengths may shape how they perceive and adopt emerging technologies. From the perspective of Hope Theory, hopeful individuals actively search for pathways that facilitate goal attainment (Snyder, 2002). Because GenAI provides immediate feedback, personalized assistance, and flexible support, hopeful students may perceive these technologies as effective pathways toward academic success. Consequently, students with higher levels of hope may be more likely to perceive generative AI as a useful learning resource and to report higher levels of technology acceptance.

### 5.3 The mediating role of self-directed learning

Consistent with H7, self-directed learning demonstrated a statistically significant indirect association between hope and learning engagement. This result is consistent with Self-directed Learning Theory, which emphasizes learners’ active responsibility for planning, monitoring, and evaluating their learning activities (Garrison, 1997).

Previous studies have demonstrated that self-directed learning positively predicts academic achievement, lifelong learning readiness, and learning engagement (Song and Hill, 2007). More recent research has suggested that self-directed learning becomes increasingly important in technology-enhanced learning environments because learners must independently regulate learning activities and evaluate information quality (Chiu et al., 2023). The present findings are consistent with this literature by indicating that self-directed learning is closely associated with students’ engagement in AI-supported learning environments.

The findings also suggest that hope and self-directed learning are positively associated. Hopeful students are more likely to establish goals, seek alternative solutions, and persist when facing difficulties (Feldman and Kubota, 2015; Gallagher, 2023). These characteristics closely correspond to the dimensions of self-directed learning described by Garrison (1997).

In AI-supported learning environments, students frequently need to decide how and when to use GenAI-generated content. Therefore, self-directed learning may represent an important behavioral characteristic associated with students’ learning engagement.

### 5.4 The serial mediating roles of generative AI acceptance and self-directed learning

Consistent with H8, generative AI acceptance was positively associated with self-directed learning. This finding is consistent with previous research suggesting that students who report higher levels of acceptance of AI-supported learning technologies also tend to demonstrate stronger autonomous learning behaviors. Recent studies suggest that GenAI technologies can facilitate learner autonomy by providing personalized recommendations, adaptive feedback, and immediate access to learning resources (Chiu et al., 2023; Dwivedi et al., 2023). Similarly, Lv et al. (2025) found that positive learning experiences with GenAI significantly enhance learners’ satisfaction and active participation in learning processes. A recent systematic review and meta-analysis further reported that GenAI positively influences students’ motivation, self-regulation, and engagement across educational contexts (Xia et al., 2025). Taken together, these findings suggest that students who report greater acceptance of generative AI may also be more likely to engage in self-directed learning.

The present study further found that generative AI acceptance and self-directed learning jointly demonstrated a statistically significant serial indirect association between hope and learning engagement. Although previous studies have separately investigated hope (Gallagher, 2023), technology acceptance (Shahzad et al., 2025; Strzelecki, 2024), and self-directed learning (Garrison, 1997), few studies have examined how these factors jointly contribute to learning engagement within a single theoretical framework. Recent GenAI research has increasingly emphasized the importance of integrating psychological, technological, and behavioral perspectives to understand educational outcomes (Dwivedi et al., 2023; Wang and Wu, 2025). Similarly, Xia et al. (2025) highlighted the need to explore the mechanisms through which GenAI influences motivation and engagement rather than focusing solely on direct effects. The present findings are consistent with this emerging perspective by suggesting that the association between hope and learning engagement is related not only to students’ psychological resources but also to their acceptance of generative AI and their self-directed learning behaviors.

Although the serial indirect association was statistically significant, its magnitude was smaller than those of the two individual indirect associations. This finding suggests that the combined pathway through generative AI acceptance and self-directed learning represents only one of several processes associated with learning engagement. From a practical perspective, improving students’ learning engagement may require simultaneous attention to psychological resources, generative AI acceptance, self-directed learning, and other contextual factors beyond those examined in the present study.

### 5.5 Theoretical and practical contributions

The present study contributes to the literature by proposing and empirically examining a psychological–technological–behavioral framework linking hope, generative AI acceptance, self-directed learning, and learning engagement. Within this framework, hope represents a positive psychological resource, generative AI acceptance reflects students’ acceptance of AI-supported learning technologies, and self-directed learning reflects learners’ active regulation of their own learning. The significant serial mediation observed in this study suggests that students’ learning engagement in AI-supported environments is associated with the combined influence of psychological resources, technology acceptance, and learning behaviors rather than any single factor operating independently.

This study makes three theoretical contributions. First, it broadens the application of Hope Theory to the context of generative AI-supported learning by showing that hope is associated with learning engagement not only directly but also indirectly through learners’ acceptance of generative AI and their self-directed learning behaviors. Rather than suggesting that hope directly determines learning engagement, the findings indicate that this association is consistent with a broader theoretical framework incorporating technology-related and behavioral factors. Second, the study contributes to the emerging literature on Generative AI Acceptance by suggesting that hope is an important psychological antecedent. The findings are broadly consistent with the technology acceptance literature, suggesting that students who perceive generative AI as useful and report stronger intentions to use it tend to demonstrate higher levels of self-directed learning. In contrast, technology anxiety represents an affective component of acceptance that may inhibit students’ willingness to adopt generative AI in educational settings. Third, by integrating psychological, technological, and behavioral perspectives within a single conceptual framework, the study provides a more comprehensive understanding of the factors associated with learning engagement in AI-supported learning environments. The substantial proportion of explained variance further suggests that these perspectives jointly contribute to understanding learning engagement.

The findings also have several practical implications for higher education. First, universities should complement the provision of generative AI technologies with initiatives that foster students’ hope, such as goal-setting activities, academic mentoring, and resilience-building programs, thereby strengthening the psychological resources that support learning. Second, educators should promote the responsible and pedagogically meaningful use of generative AI by helping students recognize its educational value while encouraging critical evaluation of AI-generated content. Third, because self-directed learning was found to be closely associated with both hope and learning engagement, instructional design should place greater emphasis on cultivating students’ self-regulation, learning planning, monitoring, and reflective evaluation skills. Integrating psychological support, AI literacy, and self-directed learning into curriculum design may better prepare students to engage effectively with AI-supported learning environments while maximizing the educational potential of generative AI.

## 6 Conclusion and limitations

This study examined the associations among hope, generative AI acceptance, self-directed learning, and learning engagement among university students by integrating Hope Theory, the Technology Acceptance Model (TAM), and Self-directed Learning Theory within a psychological–technological–behavioral framework. The findings indicated that hope was positively associated with learning engagement both directly and indirectly through generative AI acceptance and self-directed learning. In particular, the significant serial indirect association suggests that students’ psychological resources, generative AI acceptance, and self-directed learning are jointly associated with learning engagement in AI-supported learning environments. These findings contribute to the growing literature by providing an integrated perspective on how psychological, technological, and behavioral factors are associated with learning engagement in the era of generative AI.

Several limitations should be acknowledged. First, the cross-sectional design precludes conclusions regarding causal or temporal relationships among the variables. Future studies should employ longitudinal or experimental designs to further examine the proposed associations over time. Second, all data were collected through self-report measures, which may be influenced by common method bias and social desirability despite the statistical procedures used to minimize these concerns. Third, although the proposed model explained a substantial proportion of the variance in learning engagement, additional individual and contextual factors, such as AI literacy, self-efficacy, trust in AI, and instructional support, may provide a more comprehensive understanding of students’ learning engagement and should be considered in future research. Fourth, the revised measurement structure of several constructs was developed through item refinement based on exploratory and confirmatory factor analyses conducted on the same sample. Although this approach was guided by both statistical and theoretical considerations and yielded satisfactory psychometric properties, the resulting measurement model should be regarded as exploratory. Future studies should validate the revised factor structure using independent samples and cross-validation procedures.

Overall, this study suggests that learning engagement in AI-supported higher education is associated with the combined roles of psychological resources, generative AI acceptance, and self-directed learning rather than any single factor alone. The findings are consistent with the proposed psychological–technological–behavioral framework and offer theoretical insights into learning engagement in AI-supported educational contexts. They also provide practical implications for higher education institutions seeking to promote meaningful student engagement through the effective integration of generative AI and the cultivation of self-directed learning.

## Data Availability

The raw data supporting the conclusions of this article will be made available by the authors, without undue reservation.

## Supplementary material

Supplementary Table 1 CFA results, correlation analysis, discriminant validity, CMB, and SEM outputs.

Supplementary Table 2 Scale refinement details, including deleted items, factor loadings, and parceling strategy.

Supplementary Table 3 Alternative model comparisons and sensitivity analysis results.
