# Correction: The relationship between hope and learning engagement among Chinese university students: the serial mediating roles of generative AI acceptance and self-directed learning

**DOI:** 10.3389/fpsyg.2026.1979889

**Published:** 2026-09-08

**Authors:** Yinshun Zhang, Zimin Wang, Huafu Xu

**Affiliations:** 1 School of Financial Technology and Accounting, Guangdong University of Science & Technology, Dongguan, Guangdong, China; 2 Department of Convergence Lifelong Education, Hanseo University, Seosan, Republic of Korea; 3 School of Computer Science and Information Security, Guilin University of Electronic Technology, Guilin, China; 4 Graduate School of Education, Peking University, Beijing, China; 5 Guangxi Zhuang Autonomous Region Bureau of Big Data Development, Nanning, China

**Keywords:** generative AI acceptance, hope, learning engagement, self-directed learning, university student

Affiliation Guangxi Zhuang Autonomous Region Bureau of Big Data Development, Nanning, China was omitted from the paper. This affiliation has now been added for author Huafu Xu.

The original version of this article has been updated.

